# Altered amino acid profile in patients with SARS-CoV-2 infection

**DOI:** 10.1073/pnas.2101708118

**Published:** 2021-06-04

**Authors:** Chris A. Rees, Christina A. Rostad, Grace Mantus, Evan J. Anderson, Ann Chahroudi, Preeti Jaggi, Jens Wrammert, Juan B. Ochoa, Augusto Ochoa, Rajit K. Basu, Stacy Heilman, Frank Harris, Stacey A. Lapp, Laila Hussaini, Miriam B. Vos, Lou Ann Brown, Claudia R. Morris

**Affiliations:** ^a^Division of Pediatric Emergency Medicine, Boston Children’s Hospital, Harvard Medical School, Boston, MA 02115;; ^b^Department of Pediatrics, Emory University School of Medicine, Atlanta, GA 30322;; ^c^Children’s Healthcare of Atlanta, Atlanta, GA 30329;; ^d^Department of Pulmonary/Critical Care, Hunterdon Medical Center, Milford, NJ 08848;; ^e^Stanley S. Scott Cancer Center, Louisiana State University Health, New Orleans, LA 70112

**Keywords:** arginine, nitric oxide, multisystem inflammatory syndrome in children, tetrahydrobiopterin, COVID-19

## Abstract

Low plasma arginine bioavailability has been implicated in endothelial dysfunction and immune dysregulation. The role of arginine in COVID-19 is unknown, but could contribute to cellular damage if low. Our objective was to determine arginine bioavailability in adults and children with COVID-19 vs. healthy controls. We hypothesized that arginine bioavailability would be low in patients with COVID-19 and multisystem inflammatory syndrome in children (MIS-C). We conducted a prospective observational study of three patient cohorts; arginine bioavailability was determined in asymptomatic healthy controls, adults hospitalized with COVID-19, and hospitalized children/adolescents <21 y old with COVID-19, MIS-C, or asymptomatic severe acute respiratory syndrome coronavirus 2 (SARS-CoV-2) infection identified on admission screen. Mean patient plasma amino acids were compared to controls using the Student’s *t* test. Arginine-to-ornithine ratio, a biomarker of arginase activity, and global arginine bioavailability ratio (GABR, arginine/[ornithine+citrulline]) were assessed in all three groups. A total of 80 patients were included (28 controls, 32 adults with COVID-19, and 20 pediatric patients with COVID-19/MIS-C). Mean plasma arginine and arginine bioavailability ratios were lower among adult and pediatric patients with COVID-19/MIS-C compared to controls. There was no difference between arginine bioavailability in children with COVID-19 vs. MIS-C. Adults and children with COVID-19 and MIS-C in our cohort had low arginine bioavailability compared to healthy adult controls. This may contribute to immune dysregulation and endothelial dysfunction in COVID-19. Low arginine-to-ornithine ratio in patients with COVID-19 or MIS-C suggests an elevation of arginase activity. Further study is merited to explore the role of arginine dysregulation in COVID-19.

Emerging evidence suggests that endothelial dysfunction plays a role in the development of lung injury in COVID-19 in both adults and children (1, 2). Low arginine bioavailability has been implicated in the development of endothelial dysfunction and T cell dysregulation (3, 4) and contributes to the pathophysiology of multiple diseases (5). However, studies evaluating the potential role of arginine bioavailability in COVID-19 and multisystem inflammatory syndrome in children (MIS-C) are lacking. Our objective was to evaluate amino acids in hospitalized adults and children with COVID-19, hypothesizing that arginine bioavailability would be low compared to healthy controls.

## Results

A total of 80 participants were included (28 adult controls, 32 hospitalized adults with COVID-19, and 20 hospitalized children/adolescents). Patient characteristics are summarized in Table1. Within the pediatric cohort, nine had COVID-19, nine had MIS-C, and two were asymptomatic.

**Table 1.** Demographics and plasma amino acid levels in controls vs. adult and pediatric COVID-19 patients

Clinical characteristics	Controls (*n* = 28)	Adult COVID-19 (*n* = 32)	*P* value	Pediatric COVID-19/MIS-C (*n* = 20)	*P* value
Patient demographics					
Mean age ± SD (range in years)	(41−50)***	60 ± 17 (20−99)	—	11 ± 5 (3−20)	—
Female, n (%)	20 (71.4)	11 (34.3)	—	10 (50.0)	—
Comorbidity present, n (%)	4 (14.3)	17 (53.1)	—	5 (25.0)	—
Multisystem inflammatory syndrome in children, n (%)	—	—	—	9 (45.0)	—
Infiltrate on chest X-ray, n (%)	—	28 (87.5)	—	15 (75.0)	—
Supplemental oxygen, n (%)	—	19 (59.4)	—	14 (70.0)	—
Ventilator use, n (%)	—	7 (21.9)	—	3 (15.0)	—
Intensive care unit, n (%)	—	9 (28.1)	—	16 (80.0)	—
Total days in hospital, median (IQR)	—	18.25 (14−31)	—	9 (8−22)	—
Death, n (%)	—	3 (9.4)	—	2 (10.0)	—
			—		—
			—		—

Amino acids (μM)					
Arginine pathway-related amino acids and ratios					
Arginine	92.9 (29.2)	58.2 (50.8)	**0.002**	52.0 (52.3)	**0.001**
Ornithine	98.5 (30.1)	110.0 (51.3)	0.30	110.7 (65.7)	0.39
Citrulline	18.4 (4.1)	12.8 (6.9)	**<0.001**	6.9 (5.1)	**<0.001**
Glutamate	211.4 (63.7)	213.9 (132.4)	0.93	165.1 (123.3)	0.09
Glutamine	1,023.0 (163.7)	614.5 (282.2)	**<0.001**	910.6 (293.5)	0.10
Arginine-to-ornithine ratio	1.01 (0.40)	0.55 (0.46)	**<0.001**	0.52 (0.45)	**<0.001**
Global arginine bioavailability ratio	0.84 (0.31)	0.48 (0.38)	**<0.001**	0.49 (0.43)	**0.002**
Glutamine-to-glutamate ratio	5.41 (2.24)	4.69 (3.73)	0.38	8.62 (5.92)	**0.01**
Other amino acids					
Alanine	504.7 (98.0)	328.9 (114.6)	**<0.001**	331.0 (119.1)	**<0.001**
Cysteine	4.3 (1.5)	4.0 (1.4)	0.41	4.1 (2.0)	0.73
Cystine	7.0 (3.5)	7.2 (5.7)	0.87	11.7 (6.6)	**0.003**
Glycine	6,884.0 (2,197)	4,286.2 (1,195)	**<0.001**	5,806.1 (1,505)	0.06
Histidine	76.2 (14.3)	39.3 (23.5)	**<0.001**	52.9 (27.0)	**<0.001**
Isoleucine	20.2 (5.4)	18.7 (7.4)	0.38	16.0 (8.4)	0.04
Leucine	106.8 (39.2)	102.1 (63.8)	0.74	77.3 (65.5)	0.06
Lysine	231.2 (37.5)	219.0 (68.3)	0.41	202.5 (99.4)	0.17
Methionine	25.4 (7.0)	21.2 (7.9)	**0.03**	21.9 (12.6)	0.23
Phenylalanine	40.8 (7.1)	57.7 (42.7)	**0.04**	55.4 (23.4)	**0.003**
Proline	246.4 (63.4)	176.3 (65.3)	**<0.001**	175.2 (79.4)	**0.001**
Serine	267.1 (58.5)	212.7 (74.6)	**0.003**	298.0 (111.8)	0.22
Threonine	40.6 (11.3)	34.7 (13.5)	0.07	42.0 (25.1)	0.80
Tryptophan	40.8 (7.3)	21.6 (7.0)	**<0.001**	20.5 (10.7)	**<0.001**
Tyrosine	46.2 (10.1)	65.4 (38.1)	**0.01**	32.7 (11.7)	**<0.001**
Valine	190.7 (41.2)	214.6 (82.5)	0.17	167.2 (66.3)	0.14

*** Only mean age range in decades available.

**Fig. 1.**
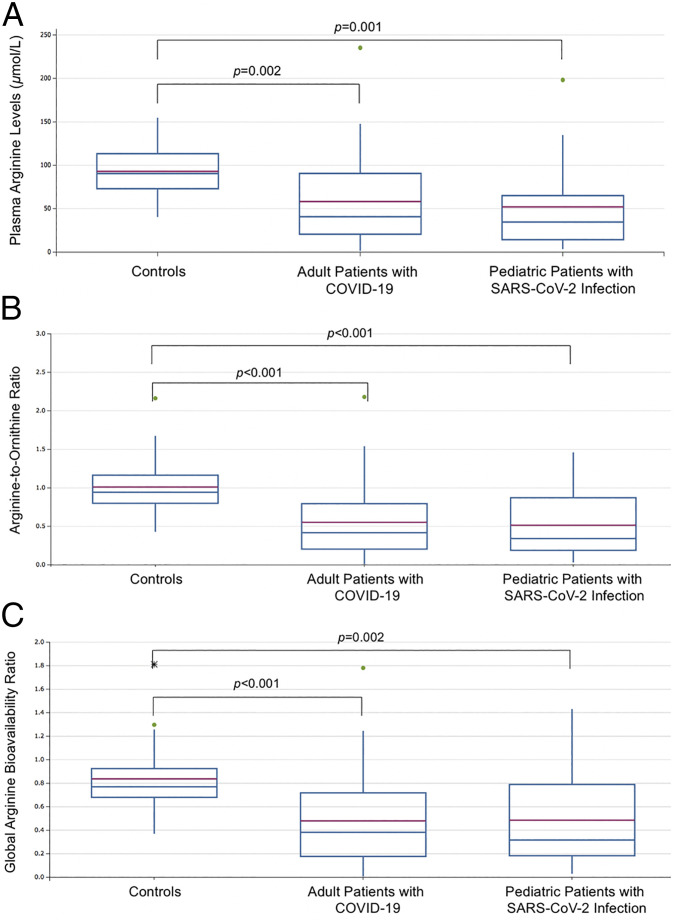
Plasma arginine bioavailability (*A*), arginine-to-ornithine ratios (*B*), and global arginine bioavailability ratios (*C*) among the three cohorts. Green dots represent outlier values. Asterisk represents an extreme outlier value.

The amino acid profiles in COVID-19 patients were abnormal (Table1). Mean plasma arginine bioavailability among controls who were seronegative for severe acute respiratory syndrome coronavirus 2 (SARS-CoV-2) IgG antibodies was similar to values among controls who had anti−SARS-CoV-2 IgG antibodies (96.8 ± 30.0 vs. 87.8 ±28.6 μmol/L, respectively; *P* = 0.43). Arginine concentration of <50 μmol/L was identified in 53.1% (17/32) of adults and 66.7% (12/20) of children with SARS-CoV-2 infection, but in only 3.6% (1/28) of controls. Arginine bioavailability, measured by arginine-to-ornithine ratio and global arginine bioavailability ratio (GABR), was significantly lower among both adults and children with SARS-CoV-2 infection compared to controls (Fig. 1). Arginine bioavailability among children with MIS-C was also significantly lower than controls (*P* < 0.001), but did not differ significantly from adults or children with COVID-19 (*P* = 0.35).

There was no correlation between plasma arginine concentration and lymphocyte count among children with SARS-CoV-2 infection (*r* = 0.23, *P* = 0.35), but there was a trend with arginine-to-ornithine ratio (*r* = 0.44, *P* = 0.07) and GABR (*r* = 0.43, *P* = 0.07).

## Discussion

Our finding of acute arginine depletion in COVID-19 among adults is consistent with findings from a recent study of 26 severely ill adults in France (6). We identify arginine depletion in both children with SARS-CoV-2 infection and those with MIS-C. Predicated on prior work describing the pathophysiology of arginine depletion (3–5), we hypothesize that arginine depletion in COVID-19 and MIS-C may contribute to endothelial dysfunction, T cell dysregulation, and coagulopathy that have been observed in COVID-19. Further studies evaluating the role of arginine in the pathophysiology of COVID-19 and MIS-C, in particular, are merited.

Low arginine-to-ornithine ratio and GABR suggest increased arginase activity and potentially impaired arginine synthesis, respectively (7), in COVID-19 and MIS-C. Furthermore, as adult controls with anti-SARS-CoV-2 antibodies who recovered from COVID-19 had normal arginine bioavailability, arginine depletion during COVID-19 appears to be transient. Although there was no correlation between arginine concentration and lymphocyte counts, a trend with arginine bioavailability ratios was observed, likely limited by a relatively small sample size. Ultimately, a majority of COVID-19 patients had low arginine levels, which has been implicated in T cell dysfunction. With arginine concentrations of <50 μM, stimulated T cells have demonstrated reduced expression of the CD3ζ chain, the primary signaling chain in the T cell receptor complex, impairing T cell proliferation and IFN-γ production (4).

A recent study proposed arginine depletion and the use of arginase therapies as potential treatments for COVID-19 (8). However, our data suggest this may potentially exacerbate COVID-19 illness, given the extant arginine depletion in COVID-19. Studies evaluating arginine as a potential therapy for COVID-19 are merited. If arginine supplementation shows promise in the treatment of COVID-19, it may serve as an additional treatment option in resource-limited settings without access to vaccines or therapies for COVID-19.

Multiple amino acid anomalies beyond arginine were noted in patients with COVID-19 that may provide insight into metabolic derangements and could have clinical consequences. Compared to controls, glutamine, an arginine precursor that becomes essential during critical illness (5), was significantly lower in adult patients with COVID-19 but not among the pediatric cohort. Tryptophan, a serotonin precursor, was reduced by nearly 50% in COVID-19 patients, which may have neuropsychiatric implications (9). Phenylalanine was high, while citrulline was low in COVID-19 patients, implicating potential dysfunction of tetrahydrobiopterin, an essential nitric oxide synthase cofactor (3, 5). Decreased energy metabolism, through both the tricarboxylic acid cycle and the citric acid cycle, is suggested by COVID-19 amino acid profiles. These data confirm metabolic derangements identified in adults with SARS-CoV-2 infection (10) that may also affect children with COVID-19 and MIS-C.

Although our observations are open avenues for potential therapies for COVID-19 and MIS-C targeting arginine augmentation, our study has limitations. We did not identify the mechanism(s) underlying arginine depletion, which potentially includes reduced protein consumption in ill patients with poor oral intake, renal dysfunction, or increased metabolism via arginase. We also did not determine the impact of low arginine bioavailability on nitric oxide production, which could be assessed in future studies by measuring flow-mediated vasodilation. We compared pediatric samples to an adult control group; however, plasma arginine concentrations in our adult samples are similar to those in pediatric controls (11). Furthermore, our relatively small sample size may have precluded our analysis reaching statistical significance, even in the presence of true biological phenomena.

## Materials and Methods

### Study Design.

We conducted a prospective observational study of three cohorts in Atlanta, GA. COVID-19−positive patients were SARS-CoV-2 positive by nasopharyngeal PCR testing. Asymptomatic, healthy controls were ≥18 y old screened for SARS-CoV-2 IgG antibodies. Those with anti−SARS-CoV-2 IgG antibodies were matched by age and gender with adults who tested negative. All controls had been asymptomatic for at least 2 wk prior to antibody testing; samples from controls with anti−SARS-CoV-2 antibodies represent recovered, convalescent COVID-19 samples. The second cohort comprised adults ≥18 y old hospitalized with COVID-19. The third cohort consisted of children/adolescents <21 y old who were hospitalized with either confirmed COVID-19, MIS-C (12), or asymptomatic SARS-CoV-2 infection identified through admission screening.

Studies were approved by the Institutional Review Board at Emory University and/or Children’s Healthcare of Atlanta. Informed consent and assent was obtained from all participants or caregivers, where appropriate. Readers may access the data via https://osf.io/vtj9w/ (DOI: 10.17605/OSF.IO/VTJ9W).

### Plasma Arginine Measurement.

Plasma amino acids were analyzed through previously described methods (13).

### Analysis.

We calculated mean (± SDs) plasma amino acids and compared each group to controls using Student’s *t* test. We calculated mean arginine-to-ornithine ratio (an indirect biomarker of arginase, the enzyme responsible for arginine conversion to ornithine) (7) and GABR among all three groups. Diminished GABR demonstrates impaired arginine synthesis in the setting of renal dysfunction (7). We determined mean glutamine-to-glutamate ratio in each group, as it is low in sickle cell disease (14), a condition of endothelial dysfunction, and elevated in neuropsychiatric conditions such schizophrenia (15). Pearson’s correlation coefficient was used to compare arginine bioavailability to lymphocyte counts. Statistical analyses were done using Microsoft Excel 2016.

## Data Availability

Raw data have been deposited in Open Science Framework, https://osf.io/vtj9w/ (DOI: 10.17605/OSF.IO/VTJ9W).
